# Facial attractiveness is only weakly linked to genome–wide heterozygosity

**DOI:** 10.3389/fpsyg.2023.1009962

**Published:** 2023-04-20

**Authors:** Martin Fieder, Susanne Huber

**Affiliations:** ^1^Department of Evolutionary Anthropology and Network of Human Evolution and Archeological Sciences (HEAS), University of Vienna, Vienna, Austria; ^2^Religion and Transformation in Contemporary Society, University of Vienna, Vienna, Austria

**Keywords:** SNP, genome wide homozygosity, MHC homozygosity, facial attractiveness, runs of homozygosity

## Abstract

**Introduction:**

It has been frequently suggested that overall genomic heterozygosity and, particularly, heterozygosity of loci on the so-called major histocompatibility complex (MHC), which is responsible for the recognition of foreign substances/ pathogens and the recognition of self and non-self, is associated with better health and better resistance to infections and parasites. It has further been speculated that such a potentially beneficial heterozygosity can be detected through body odor and facial attractiveness.

**Methods:**

On the basis of genome wide SNP data (713,014 SNPs) of participants from the Wisconsin Longitudinal Study, we therefore investigated whether homozygosity either on the MHC (measured as inbreeding coefficient) or genome-wide (measured as runs of homozygosity and inbreeding coefficient) is associated with rated facial attractiveness.

**Results:**

Although we found that the genome-wide average length of homozygous segments and the genome-wide inbreeding coefficient are significantly negatively associated with some measures of facial attractiveness, if corrected for multiple testing, any significant association was no longer formally significant after correction. In addition, the variance in facial attractiveness explained by the genome wide homozygosity is very low (<0.15%). We did not find any significant association between the inbreeding coefficient on the MHC and facial attractiveness.

**Discussion:**

We only find a weak association of genome- wide heterozygosity and facial attractiveness.

## Introduction

It has been frequently argued that fitness benefits may be gained by a preference for mates having traits that indicate “genetic makeup.” However, it remains unclear, which traits do indicate a preferable genetic makeup (Roberts et al., 2005). An important line of argument is that overall heterozygosity and, particularly, heterozygosity at certain loci may be an indicator of a preferable genetic makeup. Accordingly, it has been demonstrated that, conversely, higher genome-wide homozygosity is associated with a variety of disadvantageous traits. Clark et al. (2019), for instance, showed on the basis of 1.4 million individuals that a higher genome-wide homozygosity (measured as “runs of homozygosity” ROH) is associated with apparently deleterious changes in 32 out of 100 phenotypic traits, ranging from anthropometric measures, physiological parameters, up to mental conditions. An earlier study analyzing 354,224 individuals from 102 cohorts also reported on a significant association between total length of ROH and four complex traits, showing that increased homozygosity (equivalent to first order cousins) was correlated with lower body height, lower education, weaker cognitive ability, and forced expiratory lung volume (Joshi et al., 2015). In addition, Abdellaoui et al. (2013) showed that higher educated individuals are lower on ROH.

In particular, the so-called major histocompatibility complex (MHC) is assumed to be a genomic area where heterozygosity may confer fitness benefits as it has been shown that heterozygous MHC alleles cause higher immune competence (Havlíček et al., 2020). Accordingly, the more polymorphic loci controlling immunological recognition of foreign substances and pathogens are, the more efficient is the immune system to parry intruders. By enhancing resistance to infectious diseases, a more polymorphic MHC complex may thus confer a selection advantage, the so-called “heterozygote advantage hypothesis” (Penn et al., 2002). Indeed, genes on the MHC encode for proteins of the immune system and are among the most polymorphic loci in the whole genome. It has been theorized that selection and, particularly, sexual selection may have been the most important driver for the high polymorphism of the MHC complex (Klein and O’huigin, 1997, reviewed in Havlíček et al., 2020). Evidence for sexual selection has been found in various species (Bernatchez and Landry, 2003), showing negative (disassortative) mating for the MHC complex, i.e., both sexes prefer partners with an MHC genotype dissimilar to their own genotype. An MHC-dissimilar mating partner, in turn, increases offspring heterozygosity at the MHC, which has been suggested to be advantageous for the progeny (Kamiya et al., 2014). Accordingly, some studies have shown that MHC-heterozygosity is beneficial if the immune system is under a pathogen challenge (Penn et al., 2002). It remains unclear, however, which amount of heterozygosity is beneficial as also potential outbreeding depression has to be considered (Wegner et al., 2003). Outbreeding depression occurs whenever mating between genetically too distant individuals leads to a reduction of fitness, for instance, owing to problems during recombination (Edmands, 1999).

The detection of heterozygous as well as genetically more dissimilar mating partners may be a mechanism to ensure heterozygosity in the progeny (Roberts et al., 2005), which has been shown to provide advantages in terms of health and other traits (Clark et al., 2019). In humans, genetic dissimilarity as well as heterozygosity among mates has been investigated particularly for the MHC complex so far. Analysing results from 30 studies across seven primate species, Winternitz et al. (2017) found that in general, primates show a trend of preferring more MHC-diverse mates, but they found no systematic evidence that primates particularly choose MHC dissimilar mates. In humans, they found both a preference for MHC-dissimilar and MHC-similar mates. Additionally, analyzing results from existing primates’ studies, Winternitz and Abbate (2015) found fewer studies in support of mate selection for optimal diversity or for specific “good gene.”

It has also been speculated, how humans may recognize MHC-heterozygosity as well as genetic dissimilarity in potential mates. Two possible characteristics have been proposed: body odor (reviewed in Havliček and Roberts, 2009) and facial attractiveness (Havlíček et al., 2020), the latter in particular with regard to MHC heterozygosity (Havliček and Roberts, 2009). Particularly, it has been speculated that an association of heterozygosity at the MHC complex and facial attractiveness would render it possible to detect the heterozygosity of MHC genes and, hence, a preferable genetic makeup by facial attractiveness (Thornhill et al., 2003; Havlíček et al., 2020). It has been argued that more attractive faces may indicate a more heterozygous arrangement of genetic loci on the MHC complex, and thus a higher immune competence (Havliček and Roberts, 2009). Indeed, Lie et al. (2008) found on the basis of genotyped microsatellite markers situated within and outside the MHC, that MHC may play an important role in face preferences: MHC heterozygosity positively predicted male attractiveness, and more specifically facial averageness. In females, standardized mean d2 (mutational differences among microsatellite alleles) predicted facial symmetry and thus attractiveness (Pflüger et al., 2012). Hence facial characteristics may provide cues to genetic quality in both males and females. Lee et al. (2016), however, were not able to verify that facial attractiveness as a preferred trait in a mate is associated with “genetic quality.” They used a twin sample to estimate the heritability of facial averageness, which is usually reported of being positively associated with attractiveness. Although they found a genetic component for facial averageness and also a significant phenotypic correlation between facial averageness and attractiveness, they were not able to conclude that the genes that affect facial averageness also affect facial attractiveness.

Also results on MHC-heterozygosity advantage as well as dissimilarity in the context of mating are mixed. Using genome-wide association data from 872 European-American spouses provided by the Health and Retirement Study, Qiao et al. (2018) found no evidence for spouse’s dissimilarities on the MHC region. Also, Croy et al. (2020) did not find evidence for MHC dissimilarity in mated couples. In contrast, on the basis of 883 European and Middle Eastern couples, Dandine-Roulland et al. (2019) found that couples from Northern Europe are significantly more MHC-dissimilar than random pairs, and that MHC-dissimilarity is extreme compared to the rest of the genome.

Using 3 loci on the MHC complex, Roberts et al. (2005), reported that faces of men who are heterozygous at all 3 loci are judged by women as more attractive than faces of men who are homozygous at one, two or three loci. The authors further found that the preferences for heterozygosity were independent of the degree of MHC similarity. The results may thus provide a hint that MHC-heterozygosity is more important than MCH-dissimilarity in the mating context. Roberts et al. (2005) further reported that the skin of more heterozygous men had been perceived as healthier (Roberts et al., 2005). Thornhill et al. (2003) and Coetzee et al. (2007), however, did not find any effect of MHC-heterozygosity on attractiveness. Thus, overall, the empirical results of an association between MHC dissimilarity, heterozygosity and facial attractiveness remain ambiguous.

Most studies focus on heterozygosity/homozygosity of selected genomic regions, in particular on the MHC-complex (Wu et al., 2018). Whereas, to our knowledge, genome-wide indicators of heterozygosity/homozygosity such as inbreeding and particularly genome-wide runs of homozygosity (ROH), indicating more precisely homozygosity as well as the individual demographic and population history (Ceballos et al., 2018; Clark et al., 2019), have not been analyzed so far. Based upon the hypotheses and the ambiguous results reported so far, in the current study, we therefore aimed to analyze the association between facial attractiveness and heterozygosity both genome-wide and on the MHC complex. On the basis of a large contemporary data set, encompassing 713,014 SNPs and ~ 9,000 individuals, we investigated the association between genome-wide measures of heterozygosity such as inbreeding coefficients and runs of homozygosity (ROH) and various rated measures of facial attractiveness as well as the association between rated facial attractiveness and heterozygosity only for the MHC complex.

## Methods

### Data set “Wisconsin Longitudinal Study”

The Wisconsin Longitudinal Study (2021) is a long-term study of a random sample of men and women, who graduated from Wisconsin high schools in 1957, and their siblings. The WLS panel started out with 10,317 members from the class of 1957. A second sample of 8,734 randomly selected siblings of the original graduate panel were recruited for the study. Of these combined samples, 9,027 individuals contributed saliva for genetic analysis. In total 713,014 SNPs had been genotyped. (*The Wisconsin Longitudinal Study genetic data is sponsored by the National Institute on Aging (grant numbers R01AG009775, R01AG033285 and Ro1AG041868) and was conducted by the University of Wisconsin*). Detailed information on individual recruitment, genotyping, and quality control can be found at https://www.ssc.wisc.edu/wlsresearch/documentation/GWAS/Herd_QC_report.pdf. We used the survey question on ethnicity (white versus non-white), but also accounted for population stratification by inclusion of the first 10 principal components of the population structure provided by Wisconsin Longitudinal. Kinship was estimated by King^1^ and we removed all kin closer than third order relative. With the exception of the Genome-wide Complex Trait Analysis (please see below), we only included individuals who had been surveyed as “white” and were non-kin, totaling 2,233 men and 2,500 women, thus, only rated individuals, non-kin and whites. In total we excluded 4,294 individuals that are related first and second order, and/or are non-white and/or attractiveness rating was missing. We only included whites and non- kin in our analysis as cross-ethnic analysis of genomic data as well as including kin may lead to spurious results (Mills et al., 2020).

### Measures of homozygosity

#### Inbreeding coefficient (IBD)

As a measure for homozygosity, using Plink 1.9, we calculated, (a) the **genome wide inbreeding coefficient (IBD)**, the observed and expected autosomal homozygous genotype counts on the basis of a minimal allele frequency of 5% (MAF 0.05), as well as (b) the **inbreeding coefficient for SNPs on the MHC complex** [including 152 SNPs of the following genes (all on chromosome 6) from the MHC complex: HLA_HA, HLA_B, HLA_C, HLA_E, HLA_G, HLA_F, HLA_DNB, HLA_DOB and HLA_DOA] without restrictions to a minimal allele frequency as the number of SNPs would have been too low. As genes on the MHC complex have different functions, we divided the analysis additionally in MHC complex I (HLA_HA, HLA_B, HLA_C, HLA_E, HLA_G, HLA_F) and MHC complex II (HLA_DNB, HLA_DOB and HLA_DOA). As only 152 SNPs on the MHC complex had been genotyped, we were not able to calculate any measure of runs of homozygosity (ROH) for the MHC complex.

#### Genome-wide runs of homozygosity (ROH)

ROH is a good indicator not only for describing overall genomic homozygosity but also for the size of genomic local homozygous segments, the number of homozygous segments, and the average size of those segments. Additionally, the structure and distribution of ROH renders it possible to describe population history. ROH correlates with overall estimates of homozygosity such as the genome-wide inbreeding coefficient as well as with the inbreeding coefficient for the MHC complex. In its simplest form, ROH is calculated by moving a window of fixed size in search of stretches of consecutive homozygous SNPs over each chromosome. The algorithms estimate the proportion of completely homozygous windows that surrounds a SNP. If this proportion is higher than a threshold, the SNP is marked as being in an ROH area. A review of the significance and advantage in the realm of genomic and phenotypic associations as well as a profound description of the various methods of ROH calculation can be found in Ceballos et al. (2018).

We calculated ROH on the basis of the recommendations of Howrigan et al. (2011) by first removing all SNPs with a minimal allele frequency of 5% (MAF 0.05) and performing a “moderate SNP pruning” using a 50 SNP “window,” a 5 SNPs shift, and a VIF of 2 (variance inflation factor—the ratio of the variance of the parameters estimated including several terms to only including one term). According to Howrigan et al. (2011), moderate SNP pruning and the threshold of 0.05 MAF leads to optimized results in the subsequent calculation of ROH. MAF threshold of 0.05 and pruning resulted in a total of 133.442 SNPs, which we used for the calculations of ROH in a sliding with a threshold of 50. We performed MAF removal, pruning and ROH calculation in PLINK.^2^ (plink code ROH: plink --bfile WLS --indep 50 5 2 --out WLS_Pruned; plink --bfile WLS_Pruned_F --homozyg-window-het 0 --homozyg-snp 50 --out WLS_Pruned_F_ROH).

Runs of homozygosity calculated in Plink produces 3 different measures of genome wide ROH: total number of genome wide homozygous segments (NSEG; the count of homozygous regions according to the statistical definition above), the genome-wide summary of the length of all homozygous segments in kilo base pairs (KB), and the average length of homozygous segments (AVGKB = KB/NSEG). Distribution of the different ROH/IBD estimates is displayed in Supplementary Figure S1.

### Phenotype attractiveness

In 2004 and 2008, yearbook photos from 1957—when the WLS subjects were, on average, 18 years of age (*M* = 18.2, *SD* = 0.26)—were rated by 33 judges recruited from roughly the same cohort as the WLS participants and, thus, at the time of rating aged between 63 and 91 years (*M* = 78.5). Yearbook pictures were not standardized in terms of facial expressions and head posture and showed mostly—but not exclusively—faces (Jokela, 2009). Each yearbook photo was rated by six male and six female judges, using a photo-labeled 11-point rating scale, ranging from “not at all attractive” (1) to “extremely attractive” (11). We used only photos of individuals for which both attractiveness ratings and genotype information were available, in total the photos of 2,233 males and 2,500 females, and included the following attractiveness ratings in our analysis to cover broader aspects of “attractiveness”: (i) meanrate (Normed average coder rating); (ii) meanrat trunc [Normed average coder rating (highest and lowest dropped)]; (iii) max rate (highest rating normed); (iv) min rate (lowest rating normed); (v) meanrat mcoder (normed average coder rating—male coders only), and (vi) meanrate fcoder (normed average coder rating—female coders only). Norming was done by WLS by subtracting the mean from the original values and then dividing the resulting values by the standard deviation. Albeit all types of ratings are highly correlated (between 0.81 and 0.99), we decided to use all types of rating in the analysis, not to miss any potential significant association.

### Models calculated

#### Linear models

We regressed meanrate, meanrat trunc, max rate, min rate, meanrat mcoder, and meanrat fcoder separately on IBD, NSEG, KB, as well as AVGKB, each model controlling for year of birth, sex (1 = male, 2 = female), father’s years of schooling (surveyed 1957) to control for SES and, as recommended by WLS, the first 10 principals components of the population structure (provided by WLS on basis of the SNP data, Benjamin et al. (2021)) to control for potential confounding effects of ancestry. Variance explained by each factor was calculated using the R library “MuMIn.”

#### Genome-wide complex trait analysis (GCTA)—covariates AVGKB and IBD

GCTA estimates the total amount of phenotypic variance explained by all SNPs sequenced for the individuals included in a study, assuming that most traits are highly polygenic and, therefore, a SNP only explains a small proportion of the phenotype. Thus, the basic concept behind GCTA is to fit the effects of all SNPs as random effects by a linear mixed model. Accordingly, we used GCTA^3^ to perform a complex trait analysis, with “rated attractiveness” (meanrate normed average coder rating) as phenotype, to analyze the variance explained by 572.470 SNPs (MAF > 0.05) and the two covariates (i) AVGKB and (ii) IBD, respectively, with birth year, education of father (frequencies of fathers’ education provided in Supplementary Table S2), and the 10 principal components of population structure included as quantitative covariates, and sex included as categorical covariate. We used the two covariates, AVGKB and IBD, in the GCTA models as those are the most significant homozygosity descriptors for attractiveness as indicated by the previously calculated linear models. As GCTA builds a genetic relationship matrix and, therefore, accounts profoundly for kinship, in this analysis we included also kin, in total 3,675 men and 4,120 women.

#### Ethics statement

All ethics concerning the participants of the Wisconsin Longitudinal study is with the “Wisconsin Longitudinal Study,” produced and distributed by the University of Wisconsin with funding from the National Institute of Aging (Grant numbers R01AG009775, R01AG033285, and Ro1AG041868).” Data use agreement from the 30. May 2019 between the boards of regents of the University of Wisconsin-Madison, Wisconsin Longitudinal Study (WLS) and the University of Vienna (receiving agency), wherein Martin Fieder is the researcher responsible for using the data files in the project entitled “Genome Wide Association Study and Runs of Homozygosity.” All participants provided informed written consent under a protocol approved by the Institutional Review Board of the University of Wisconsin-Madison. The status of the informed consent has been documented in detail by Wisconsin Longitudinal,^4^ only individuals with a full consent are included in the data realize. Details can be found at: https://www.ssc.wisc.edu/wlsresearch/documentation/GWAS/.

## Results

Regressing the mean rating of attractiveness on IBD for the MHC complex only, we find no significant association (estimate = 0.077, *p* = 0.29), IBD for the MHC complex explaining only a very small proportion of the variance (amount variance explained between 0.0005 and 0.084%). The same holds true for other measures of attractiveness (see Supplementary Table S1). We also find no significant association if we split between MHCI and MHCII. For both genomic regions, the association with all measurements of attractiveness remain non-significant.

Genome-wide, in the linear models, AVGKB and IBD are significantly, respectively, marginally significantly associated with most of the different indicators of the attractiveness rating, except the association of max rate and mean rate women with IBD (Table 1). The association is always negative, indicating that a higher ROH is associated with lower attractiveness rating. Though generally variance explained is very low even for the significant associations. In addition, if we applied a correction for multiple testing (e.g., Bonferroni correction would shift *p* < 0.05 to *p* < 0.0083), no estimate is significant anymore. Education of the father is significantly positively associated with the different indicators of the attractiveness rating, and being male is significantly negatively associated with female rating only (Table 1). Birth year shows no significant associations. The highest proportion of variance is explained by father’s education, which, although being rather small, is still roughly 5–10 times higher than variance explained by any of the indicators of ROH or IDB. Sex also only explains very little of the overall variance in the models. As the estimates for birth year, sex, and education of the father are very similar in each of the different models, only the estimates from the “NSEG”-model are shown (Table 1).

**Table 1:** Results of the separate linear models of mean rate, mean rate trunc, max rate, min rate, mean rate men and mean rate women, respectively, regressing on birth year, sex, education of the father, AVGKB, KB, NSEG, and IBD.

	Birth year^1^ estimate	Sex^1^ estimate	Educ father^1^ estimate	AVGKB estimate	KB estimate	NSEG estimate	IBD estimate
Mean rate	−0.007	−0.0813	0.043***	−0.00004*	−0.000003	−0.0206	−7.1047*
Mean rate trunc	−0.009	−0.0894	0.0452***	−0.00004*	−0.000003	−0.0210	−7.4237*
Max rate	0.021	−0.0610	0.036***	−0.00004.	−0.000002	−0.017	−4.5102
Min rate	−0.007	−0.0210	0.030***	−0.00005*	−0.000003	−0.020	−6.58297.
Mean rate men	0.0059	−0.096*	0.048***	−0.00004*	−0.000004	−0.023	−8.163*
Mean rate women	−0.0190	−0.067	0.039***	−0.0000.	−0.000003	−0.018	−6.042
% variance explained	0.0002–0.0032%	0.015–0.11%	0.55–1.37%	0.11–0.14%	0.008–0.069%	0.0076–0.077%	0.015–0.1%

^1^Only estimates of the regression model including NSEG are shown. *p* < 0.1, *P < 0.05, **p < 0.01, ***p < 0.001. Significant associations are in bold.

### Genome-wide complex trait analysis

In the GCTA, both the covariates AVGKB and IBD are significantly negatively associated with mean attractiveness rate, but again explain only a small proportion of the phenotypic variance (Table 2).

**Table 2:** Mean attractiveness regressing on the SNPs with MAF > 0.5, with IBD, sex, birth year and father’s education as well as the first 10 principal components of population structure.

Predictor	Phenotype	Beta	Beta (SE)	% Variance expl.	*P*
AVGKB	Mean attract	−0.000036	0.000016	0.137	0.024
IBD	Mean attract	−7.153	3.288	0.181	0.030

Values for non-significant indicators and covariates are not shown.

We further investigated the association of facial attractiveness and homozygosity around ± 30,000 bp of the two SNPs found of being associated with facial attractiveness by Hu et al. (2019) (i.e., rs2999422 located in an intron of pseudogene ANTXRLP1 and rs10165224 located in an intergenic region between protein-coding gene CDC42EP3 and RNA gene LINC00211), but did not found any significant association.

## Discussion

For the MHC complex, we found no significant association between homozygosity measured as IBD and any indicator of attractiveness rating. Hence, based on our results, we are not able to confirm the frequently discussed heterozygote advantages on the MHC complex (please see introduction), neither for the whole MHC complex, nor separately for MHCI or MHCII complex. The reasons for this null result could be various, possibly the number of cases and/or the number of SNPs on the MHC complex were too low. Whereas genome wide, with two exceptions, a significant (respectively marginal significant) negative association between the average length of homozygous segments (AVGKB) and various indicators of attractiveness ratings was found. Also, IBD was significantly negatively associated with some indicators of attractiveness, indicating that higher genome wide homozygosity is associated with lower attractiveness. Though, if corrected for multiple testing, none of these associations remained significant. In addition, the total variance explained by the different indicators of ROH and IDB was very low, ranging from 0.01 to 0.13%. Furthermore, cultures with a higher pathogen prevalence may exhibit a stronger link between MHC heterozygosity and attractiveness, as signaling heterozygosity could be more important. However, as we have no data this must remain purely speculative for the moment.

As the genome wide analysis did come up at least with some (weak) results, the following discussion focuses mainly on genome-wide measures of homozygosity/heterozygosity. In addition, according to Ceballos et al. (2018), using the whole genome accounts much better for genomic history of populations and individuals. Although the complex trait analysis (GCTA) confirms the results from the regression analysis, showing that average length of homozygous segments (AVGKB) as well as IBD are significantly negatively associated with mean attractiveness, the proportion of variance explained is very low. Albeit also explaining only a small proportion of the overall variance in attractiveness, from all analyzed variables, education of the father was the most significant predictor, indicating that socio-economic status experienced in early life affects later attractiveness (Huber and Fieder, 2014).

From our findings we conclude that, although genome-wide we find an association between higher homozygosity and lower attractiveness, effect sizes are rather small and not robust if corrected for multiple testing (see also Zaidi et al., 2019). Accordingly, to detect more substantial influences, a much larger samples size would be needed (between 12,000 and 65,000 individuals, Keller et al., 2011). We meanwhile know that attractiveness has a moderate inherited component as shown by a very recent genome wide association study (Hu et al., 2019). This Genome Wide Association Study (GWA) on basis of the WLS data set also indicates that facial attractiveness is at least moderately heritable, depending on who has been rating: heritability for attractiveness was 10.9% and non-significant if rated by females but 27.7% and significant if rated by males (Hu et al., 2019). Thus, albeit attractiveness is to some extent heritable, compared to other inherited parts of attractiveness, with a variance explained between 0.008 and 0.14%, the effect of homozygosity seems rather weak and contributes only to a small amount to the heritable component of attractiveness. As we did not find any significant association of facial attractiveness and homozygosity around the two SNPs found of being associated with facial attractiveness by Hu et al. (2019) (rs2999422, s10165224), also homozygosity around these loci does not contribute significantly to facial attractiveness.

Our results may be influenced by the negative association of parents’ SES and facial attractiveness, which is presumably caused by worse early life conditions if parents’ SES is low (Huber and Fieder, 2014). In line with this, all measures of attractiveness are highly significantly but only weakly negatively correlated with father’s education (R between 0.029 and 0.12; *p* < 0.0001). Hence, facial attractiveness may not be influenced directly by homozygosity but indirectly *via* the effect of parent’s homozygosity on father’s education (SES), and parents’ SES may in turn affect facial attractiveness of the children. As we do not have any data on homozygosity of parents, we cannot directly test this assumption. As for AVGKB, only a marginally significant negative association with father’s education did appear, we cannot exclude a link *via* parent’s SES although we have no further confirmation. We are not able to draw any final conclusions on causalities regarding the association between parent’s SES and facial attractiveness as it is, for instance, possible that genes which are associated with lower SES (Hill et al., 2019) are also associated with a lower facial attractiveness.

Overall our findings demonstrate, if at all, only a weak association between increased homozygosity and attractiveness on the genome-wide level. Even though these results indicate that genome-wide homozygosity/heterozygosity may possibly be detectable by attractiveness to a very small extent. From our results, we are neither able to confirm nor decline hypotheses suggesting that homozygosity/heterozygosity particularly on immune genes and, thus, vulnerability to pathogens, can be detected in a potential mate on the basis of facial attractiveness.

Many human small-scale populations are highly homozygous (Ceballos et al., 2018), indicating that consanguinity may have been prevalent during evolution. As a consequence, within such small-scale populations, all individuals have been homozygous to a comparable extent, rendering detecting homozygosity by attractiveness hard or even impossible. This view seems reasonable as even in our contemporary, genetically more diverse US-sample, effects of homozygosity on attractiveness are very subtle. In genetically more homogeneous populations, effects are presumably still much subtler. In order to finally confirm or reject the hypothesis that homozygosity is associated with facial attractiveness, according to Keller et al. (2011), in modern out-bred populations, a sample size between 12,000 and 65,000 individuals is needed to detect effects reliably. As to our knowledge no such big data sets exist that provide rating of attractiveness as well as genomic data, conclusions must remain provisionally for now.

## Limitations of the study

We do not have any replication sample, and attractiveness was measured on the basis of high-school yearbook photos. Also, makeup, hairstyles, the angle at which the face is photographed and other factors may have significantly influenced facial attractiveness. Furthermore, albeit rating was consistent among raters, rating took place more than 40 years after the photos had been taken. In addition, as raters and the rated individuals all lived in the same state and belonged to the same racially homogeneous population, it is not possible to generalize results over time, generations, geography or race; this holds true for the genome-wide analysis as well as the analysis of the MHC complex. Thus, as mentioned above, for the future a much larger data set across cultures will be needed in order to confirm or reject the hypotheses regarding an association of homozygosity and attractiveness.

## Data availability statement

Publicly available datasets were analyzed in this study. Genomic data are available after IRB approval by Wisconsin Longitudinal. Data can be found at: https://www.ssc.wisc.edu/wlsresearch/.

## Ethics statement

All ethics concerning the participants of the Wisconsin Longitudinal study is with the “Wisconsin Longitudinal Study”; produced and distributed by the University of Wisconsin with funding from the National Institute of Aging (Grant Numbers R01AG009775, R01AG033285, and Ro1AG041868)”. Ethical approval for the study was received by the IRB Board of the University of Vienna, Project Number 00409: Phenotypic Associations of Homozygosity. The patients/participants provided their written informed consent to participate in this study.

## Author contributions

MF and SH: study design and manuscript preparation. MF: data analysis. All authors contributed to the article and approved the submitted version.

## Funding

This research uses data from the Wisconsin Longitudinal Study, funded by the National Institute on Aging (R01 AG009775; R01 AG033285; R01 AG060737; R01 AG041868).

## Conflict of interest

The authors declare that the research was conducted in the absence of any commercial or financial relationships that could be construed as a potential conflict of interest.

## Publisher’s note

All claims expressed in this article are solely those of the authors and do not necessarily represent those of their affiliated organizations, or those of the publisher, the editors and the reviewers. Any product that may be evaluated in this article, or claim that may be made by its manufacturer, is not guaranteed or endorsed by the publisher.

## Footnotes

^1^ https://people.virginia.edu/~wc9c/KING/

^2^ http://zzz.bwh.harvard.edu/plink/

^3^ https://cnsgenomics.com/software/gcta/#Overview

^4^ https://www.ssc.wisc.edu/wlsresearch/documentation/waves/?wave=descriptive&module=dscrMod

## References

[ref1] Abdellaoui A. Hottenga J.-J. Xiao X. Scheet P. Ehli E. A. Davies G. E.. (2013). Association between autozygosity and major depression: Stratification due to religious assortment. Behav. Genet. 43, 455–467. doi: 10.1007/s10519-013-9610-1, PMID: 23978897 PMC3827717

[ref2] Benjamin D. Cesarini D. Okbay A. Turley P. (2021). Polygenic index repository user guide (version 1.0). Wisconsin Longitudinal.

[ref3] Bernatchez L. Landry C. (2003). MHC studies in nonmodel vertebrates: What have we learned about natural selection in 15 years? J. Evol. Biol. 16, 363–377. doi: 10.1046/j.1420-9101.2003.00531.x, PMID: 14635837

[ref4] Ceballos F. C. Joshi P. K. Clark D. W. Ramsay M. Wilson J. F. (2018). Runs of homozygosity: Windows into population history and trait architecture. Nat. Rev. Genet. 19, 220–234. doi: 10.1038/nrg.2017.109, PMID: 29335644

[ref5] Clark D. W. Okada Y. Moore K. H. Mason D. Pirastu N. Gandin I.. (2019). Associations of autozygosity with a broad range of human phenotypes. Nat. Commun. 10, 1–17. doi: 10.1038/s41467-019-12283-6 31673082 PMC6823371

[ref6] Coetzee V. Barrett L. Greeff J. M. Henzi S. P. Perrett D. I. Wadee A. A. (2007). Common HLA alleles associated with health, but not with facial attractiveness. PLoS One 2:e640. doi: 10.1371/journal.pone.0000640, PMID: 17653267 PMC1919430

[ref7] Croy I. Ritschel G. Kreßner-Kiel D. Schäfer L. Hummel T. Havlíček J.. (2020). Marriage does not relate to major histocompatibility complex: A genetic analysis based on 3691 couples. Proc. R. Soc. B 287:20201800. doi: 10.1098/rspb.2020.1800, PMID: 33023409 PMC7657850

[ref8] Dandine-Roulland C. Laurent R. Dall’Ara I. Toupance B. Chaix R. (2019). Genomic evidence for MHC disassortative mating in humans. Proc. R. Soc. B 286:20182664. doi: 10.1098/rspb.2018.2664, PMID: 30890093 PMC6452061

[ref9] Edmands S. (1999). Heterosis and outbreeding depression in interpopulation crosses spanning a wide range of divergence. Evolution 53, 1757–1768. doi: 10.2307/2640438, PMID: 28565458

[ref10] Havliček J. Roberts S. C. (2009). MHC-correlated mate choice in humans: A review. Psychoneuroendocrinology 34, 497–512. doi: 10.1016/j.psyneuen.2008.10.007, PMID: 19054623

[ref11] Havlíček J. Winternitz J. Roberts S. C. (2020). Major histocompatibility complex-associated odour preferences and human mate choice: Near and far horizons. Philos. Trans. R. Soc. B 375:20190260. doi: 10.1098/rstb.2019.0260, PMID: 32306884 PMC7209936

[ref12] Hill W. D. Davies N. M. Ritchie S. J. Skene N. G. Bryois J. Bell S.. (2019). Genome-wide analysis identifies molecular systems and 149 genetic loci associated with income. Nat. Commun. 10, 1–16. doi: 10.1038/s41467-019-13585-5 31844048 PMC6915786

[ref13] Howrigan D. P. Simonson M. A. Keller M. C. (2011). Detecting autozygosity through runs of homozygosity: A comparison of three autozygosity detection algorithms. BMC Genomics 12, 1–15. doi: 10.1186/1471-2164-12-460 PMC3188534 21943305

[ref14] Hu B. Shen N. Li J. J. Kang H. Hong J. Fletcher J.. (2019). Genome-wide association study reveals sex-specific genetic architecture of facial attractiveness. PLoS Genet. 15:e1007973. doi: 10.1371/journal.pgen.1007973, PMID: 30946739 PMC6448826

[ref15] HuberS.FiederM. (2014). Effects of parental socio-economic conditions on facial attractiveness. Evol. Psychol. 12:147470491401200. doi: 10.1177/14747049140120051425548886

[ref16] Jokela M. (2009). Physical attractiveness and reproductive success in humans: Evidence from the late 20th century United States. Evol. Hum. Behav. 30, 342–350. doi: 10.1016/j.evolhumbehav.2009.03.006, PMID: 21151758 PMC3000557

[ref17] Joshi P. K. Esko T. Mattsson H. Eklund N. Gandin I. Nutile T.. (2015). Directional dominance on stature and cognition in diverse human populations. Nature 523, 459–462. doi: 10.1038/nature14618, PMID: 26131930 PMC4516141

[ref18] Kamiya T. O’Dwyer K. Westerdahl H. Senior A. Nakagawa S. (2014). A quantitative review of MHC-based mating preference: The role of diversity and dissimilarity. Mol. Ecol. 23, 5151–5163. doi: 10.1111/mec.12934, PMID: 25251264

[ref19] Keller M. C. Visscher P. M. Goddard M. E. (2011). Quantification of inbreeding due to distant ancestors and its detection using dense single nucleotide polymorphism data. Genetics 189, 237–249. doi: 10.1534/genetics.111.130922, PMID: 21705750 PMC3176119

[ref20] Klein J. O’Huigin C. (1997). “MHC polymorphism and parasites” in Infection, polymorphism and evolution. eds. Hamilton W. D. Howard J. C. (Berlin: Springer), 81–88.

[ref21] Lee A. J. Mitchem D. G. Wright M. J. Martin N. G. Keller M. C. Zietsch B. P. (2016). Facial averageness and genetic quality: Testing heritability, genetic correlation with attractiveness, and the paternal age effect. Evol. Hum. Behav. 37, 61–66. doi: 10.1016/j.evolhumbehav.2015.08.003, PMID: 26858521 PMC4743547

[ref22] Lie H. C. Rhodes G. Simmons L. W. (2008). Genetic diversity revealed in human faces. Evolution 62, 2473–2486. doi: 10.1111/j.1558-5646.2008.00478.x 18691260

[ref23] Mills M. C. Barban N. Tropf F. C. (2020). An introduction to statistical genetic data analysis Boston MA: Mit Press.

[ref24] Penn D. J. Damjanovich K. Potts W. K. (2002). MHC heterozygosity confers a selective advantage against multiple-strain infections. Proc. Natl. Acad. Sci. U. S. A. 99, 11260–11264. doi: 10.1073/pnas.162006499, PMID: 12177415 PMC123244

[ref25] Pflüger L. S. Oberzaucher E. Katina S. Holzleitner I. J. Grammer K. (2012). Cues to fertility: Perceived attractiveness and facial shape predict reproductive success. Evol. Hum. Behav. 33, 708–714. doi: 10.1016/j.evolhumbehav.2012.05.005

[ref26] Qiao Z. Powell J. E. Evans D. M. (2018). MHC-dependent mate selection within 872 spousal pairs of European ancestry from the health and retirement study. Genes 9:53. doi: 10.3390/genes9010053, PMID: 29361785 PMC5793204

[ref27] Roberts S. C. Little A. C. Gosling L. M. Perrett D. I. Carter V. Jones B. C.. (2005). MHC-heterozygosity and human facial attractiveness. Evol. Hum. Behav. 26, 213–226. doi: 10.1016/j.evolhumbehav.2004.09.002

[ref28] The Wisconsin Longitudinal Study. (2021). Produced and distributed by the University of Wisconsin with funding from the National Institute of aging (Grant numbers R01AG009775, R01AG033285 and Ro1AG041868).

[ref29] Thornhill R. Gangestad S. W. Miller R. Scheyd G. McCollough J. K. Franklin M. (2003). Major histocompatibility complex genes, symmetry, and body scent attractiveness in men and women. Behav. Ecol. 14, 668–678. doi: 10.1093/beheco/arg043

[ref30] Wegner K. M. Kalbe M. Kurtz J. Reusch T. B. Milinski M. (2003). Parasite selection for immunogenetic optimality. Science 301:1343. doi: 10.1126/science.1088293, PMID: 12958352

[ref31] Winternitz J. C. Abbate J. L. (2015). Examining the evidence for major histocompatibility complex-dependent mate selection in humans and nonhuman primates. Res. Rep. Biol. 6, 73–88. doi: 10.2147/RRB.S58514

[ref32] Winternitz J. Abbate J. Huchard E. Havlíček J. Garamszegi L. (2017). Patterns of MHC-dependent mate selection in humans and nonhuman primates: A meta-analysis. Mol. Ecol. 26, 668–688. doi: 10.1111/mec.13920, PMID: 27859823

[ref33] Wu K. Chen C. Moyzis R. K. Nuno M. Yu Z. Greenberger E. (2018). More than skin deep: Major histocompatibility complex (MHC)-based attraction among Asian American speed-daters. Evol. Hum. Behav. 39, 447–456. doi: 10.1016/j.evolhumbehav.2018.04.001

[ref34] Zaidi A. A. White J. D. Mattern B. C. Liebowitz C. R. Puts D. A. Claes P.. (2019). Facial masculinity does not appear to be a condition-dependent male ornament and does not reflect MHC heterozygosity in humans. Proc. Natl. Acad. Sci. U. S. A. 116, 1633–1638. doi: 10.1073/pnas.1808659116, PMID: 30647112 PMC6358690

