# Supplementary material for: Facial attractiveness is only weakly linked to genome–wide heterozygosity
Source: Front Psychol. 2023 Apr 20;14:1009962. doi: 10.3389/fpsyg.2023.1009962 (PMC10157054; doi:10.3389/fpsyg.2023.1009962)
Supplement: Supplementary file 1 [file Data_Sheet_1.docx]

**Supplementary material**

Table S1) The different indicators of attractiveness regressing on IBD for the MHC complex, corrected for sex, birth years and father’s education. Only whites no kin.

|  | **estimates Inbreed MHC** | | **sex** | | **birth year** |  | **Father Education** | |
| --- | --- | --- | --- | --- | --- | --- | --- | --- |
| **mean rate** | 0.077 |  | -0.084 | . | -0.06 |  | 0.043 | *** |
| **mean rate trunc** | 0.077 |  | -0.092 | * | -0.008 |  | 0.045 | *** |
| **max rate** | 0.092 |  | -0.063 |  | 0.022 |  | 0.036 | *** |
| **min rate** | 0.073 |  | -0.023 |  | -0.006 |  | 0.03 | *** |
| **mean rate men** | 0.019 |  | -0.098 |  | 0.007 |  | 0.05 | *** |
| **mean rate women** | 0.137 |  | -0.07 |  | -0.018 |  | 0.039 | *** |
| **% Variance Explained** | (0.0005% - 0.084%) | | 0.015%-0.11% |  | 0.0002%- 0.0032% | | 0.55%-1.37% |  |

Table S2) Father’ years of schooling.

|  | **Frequ.** | **%** |
| --- | --- | --- |
| no high school | 1351 | 24.02 |
| attended high school | 621 | 11.04 |
| grad from hgh school | 713 | 12.68 |
| attend trde/bus schl | 290 | 5.16 |
| attended college | 210 | 3.73 |
| grad from college | 259 | 4.61 |
| has masters or ph.d. | 93 | 1.65 |


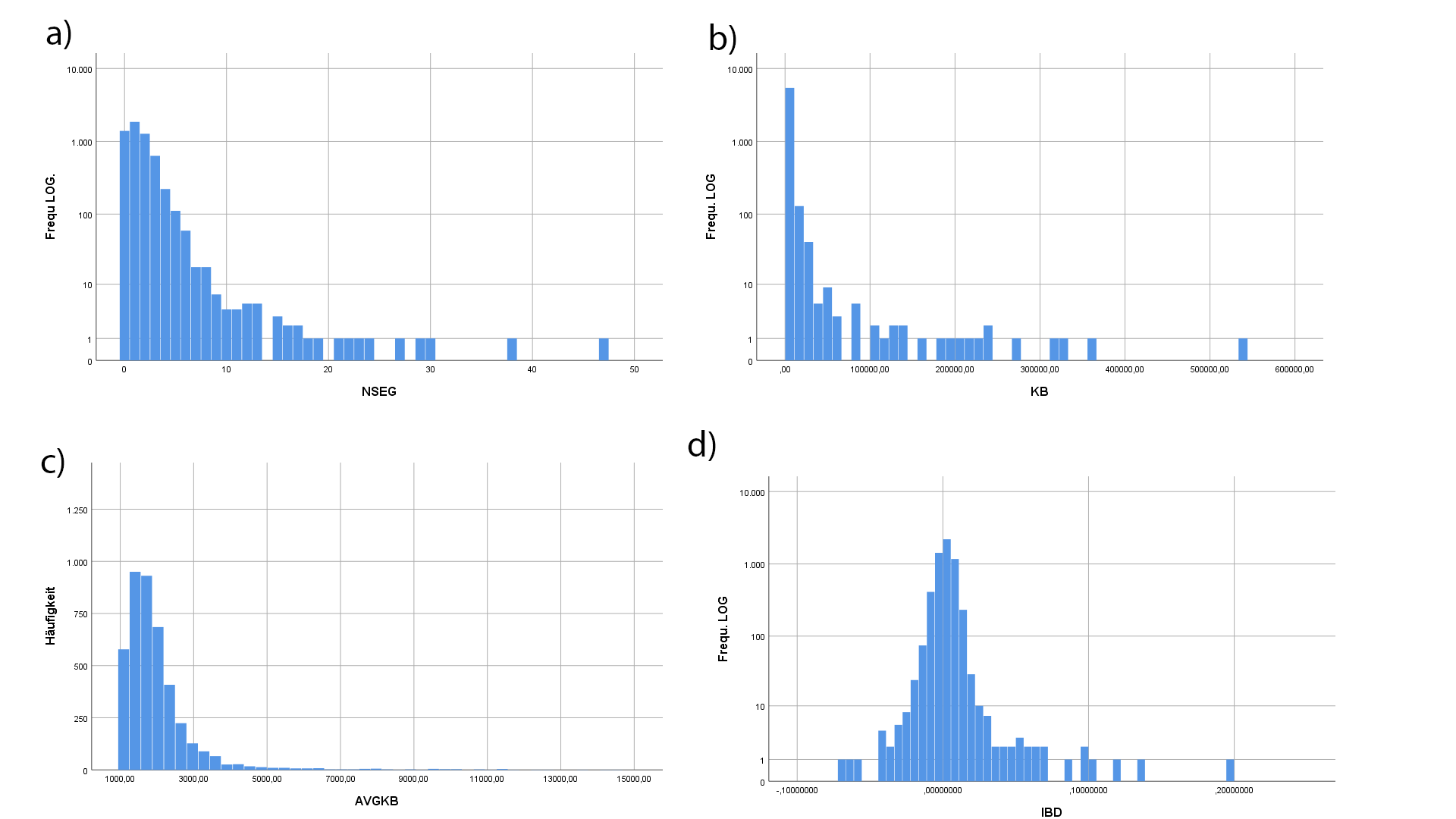


Figure S1) Distribution of the genome wide indicators for homozygosity a) NSEG, b) KB), c) AVGKB, d) IBD
